# Development of a highly pulmonary metastatic orthotopic renal cell carcinoma murine model

**DOI:** 10.1242/bio.058566

**Published:** 2021-04-20

**Authors:** Jee Soo Park, Myung Eun Lee, Seung Hwan Kim, Won Sik Jang, Won Sik Ham

**Affiliations:** Department of Urology and Urological Science Institute, Yonsei University College of Medicine, Seoul 03722, Korea

**Keywords:** Renal cell carcinoma, Metastasis, Kidney, Orthotopic, Lung, Mouse

## Abstract

The incidence of renal cell carcinoma (RCC) is high, and its outcomes remain poor. Mortality is attributable largely to metastatic disease and a dearth of effective therapeutic interventions. The lungs are the most common metastatic site. To elucidate the biological mechanisms underlying pulmonary metastasis and identify superior therapeutic strategies, we developed a novel and clinically relevant murine RCC model exhibiting enhanced pulmonary metastasis. Mice underwent intrarenal implantation using luciferase-expressing Renca, a murine renal adenocarcinoma cell line. Primary renal tumor progression and development of metastatic lung lesions were monitored in live mice using bioluminescent imaging, followed by post-mortem organ assessment. Cells were isolated from pulmonary metastases for reimplantation, followed by repeat monitoring and assessment. This process was repeated once more for a total of two *in vivo* passages to select for pulmonary metastatic Renca cell subpopulations. However, a single round of *in vivo* selection was sufficient to produce a near-maximally metastatic subpopulation. Relative to Renca cell-implanted mice, subpopulation-implanted mice exhibited shorter implantation-metastasis intervals (5 days), shorter implantation-moribundity intervals (sacrificed at 18.6±2.9 versus 22.3±1.1 days), a higher number of metastatic lung lesions at 23 days (183.9±39.0 versus 172.6±38.2) and poorer survival. Implantation of cells derived from the second round of *in vivo* selection produced no further significant differences in the above metrics. This model consistently and efficiently recapitulates RCC pulmonary metastasis while allowing *in vivo* monitoring of tumor progression, thereby facilitating elucidation of the molecular mechanisms underlying pulmonary metastasis and evaluation of therapeutic modalities.

## Abstract (precis)

**Summary:** We have developed renal cell carcinoma murine model that consistently and efficiently recapitulates pulmonary metastasis while allowing *in vivo* monitoring of tumor progression.

## INTRODUCTION

Renal cell carcinoma (RCC) is one of the top ten most frequently diagnosed cancers, with a global incidence of approximately 400,000 cases (Siegel et al., 2019). At the time of initial diagnosis, almost 30% of patients present with metastatic RCC (mRCC), whereas approximately 30% of patients presenting with localized RCC experience recurrence after treatment (Czarnecka et al., 2014). The most common sites of RCC metastases are the lungs, bone, liver, and brain (Zekri et al., 2001). Despite significant improvements in the treatment of advanced RCC over the past 30 years, including targeted therapies and immunotherapies, RCC-related mortality continues to increase, attributable largely to metastatic disease (Dutcher et al., 2020; Hollingsworth et al., 2006). Advanced RCC patients exhibit progression-free and overall survival of less than two years in the majority of cases (Acquavella and Fojo, 2013; Lee-Ying et al., 2014; Rini, 2010).

Therefore, further research is required to identify and develop more effective mRCC treatment strategies. Given that the lungs are the most common metastatic site (accounting for up to 60% of metastases) (Motzer et al., 1996), it is imperative to develop preclinical models capable of facilitating elucidation of pulmonary mRCC biological mechanisms and identification of more effective treatment strategies with superior toxicity profiles. Modern immunotherapies inhibit programmed cell death protein 1 (PD-1)-, programmed death-ligand 1 (PD-L1)-, or cytotoxic T-lymphocyte-associated protein 4 (CTLA-4)-based immune checkpoints. This ameliorates immune evasion by tumor cells largely without significant adverse effects (Motzer et al., 2018, 2019; Rini et al., 2019). Evaluation of such therapies within the present context requires hosts with intact and functional immune systems. We therefore developed a murine RCC model based on the well-described Renca cell line, originally derived from BALB/c mouse spontaneous renal adenocarcinoma (Salup and Wiltrout, 1986).

While most studies inject Renca cells subcutaneously to produce easily assessable local tumors, such an approach lacks anatomical relevance to RCC. In addition, intravenous injection of Renca cells establishes a pulmonary tumor burden, but does not allow for observation of migration from the primary to the secondary tumor site to investigate metastatic pathogenesis. Lack of an appropriate pulmonary mRCC animal model (i.e. one that mimics the tumor microenvironment, infiltration into local vasculature, and organ-tumor cellular communication (Murphy et al., 2017)) contributes to the high failure rate of novel anticancer drugs in this context (Ocana et al., 2011).

A model simultaneously exhibiting both primary and metastatic disease would facilitate crucial research examining the progression of mRCC to identify and evaluate appropriate treatment strategies. This is particularly relevant when considering that mRCC patients typically display pulmonary metastases, and that metastases are the major cause of mortality in these patients. The present study implants luciferase-expressing Renca cells (Renca/luc) and selects a highly pulmonary metastatic subset [Renca(HM)/luc] for reimplantation, thereby establishing a highly efficient and consistent murine model of pulmonary mRCC that recapitulates major aspects of human mRCC. This provides a more suitable model for the *in vivo* study of this cancer and its treatment.

## RESULTS

Briefly, Renca/luc cells were orthotopically implanted into the kidneys of an initial set of mice via injection. Progression of RCC (including metastasis) was evaluated at intervals via bioluminescent imaging (BLI). After sacrifice, renal and pulmonary tumor burdens were evaluated, and Renca(HM)/luc cells were isolated from pulmonary metastases for reimplantation into a second set of mice. Tumor progression and organ tumor burdens were again evaluated. After the mice were euthanized, pulmonary metastatic cells were again isolated for reimplantation into a third set of mice, and identical evaluations were repeated a final time. In order to verify that cells from the kidney tumors and pulmonary metastases originated from same Renca/luc cells that were injected, we have checked the morphology of cells from both kidney tumors and pulmonary metastases that represented to be identical (Fig. S1). Moreover, we could simultaneously detect BLI signal intensity from both the kidney and lung fields, supporting that Renca/luc cells which was implanted to the kidney were metastasized to the lung.

**Fig. 1.**
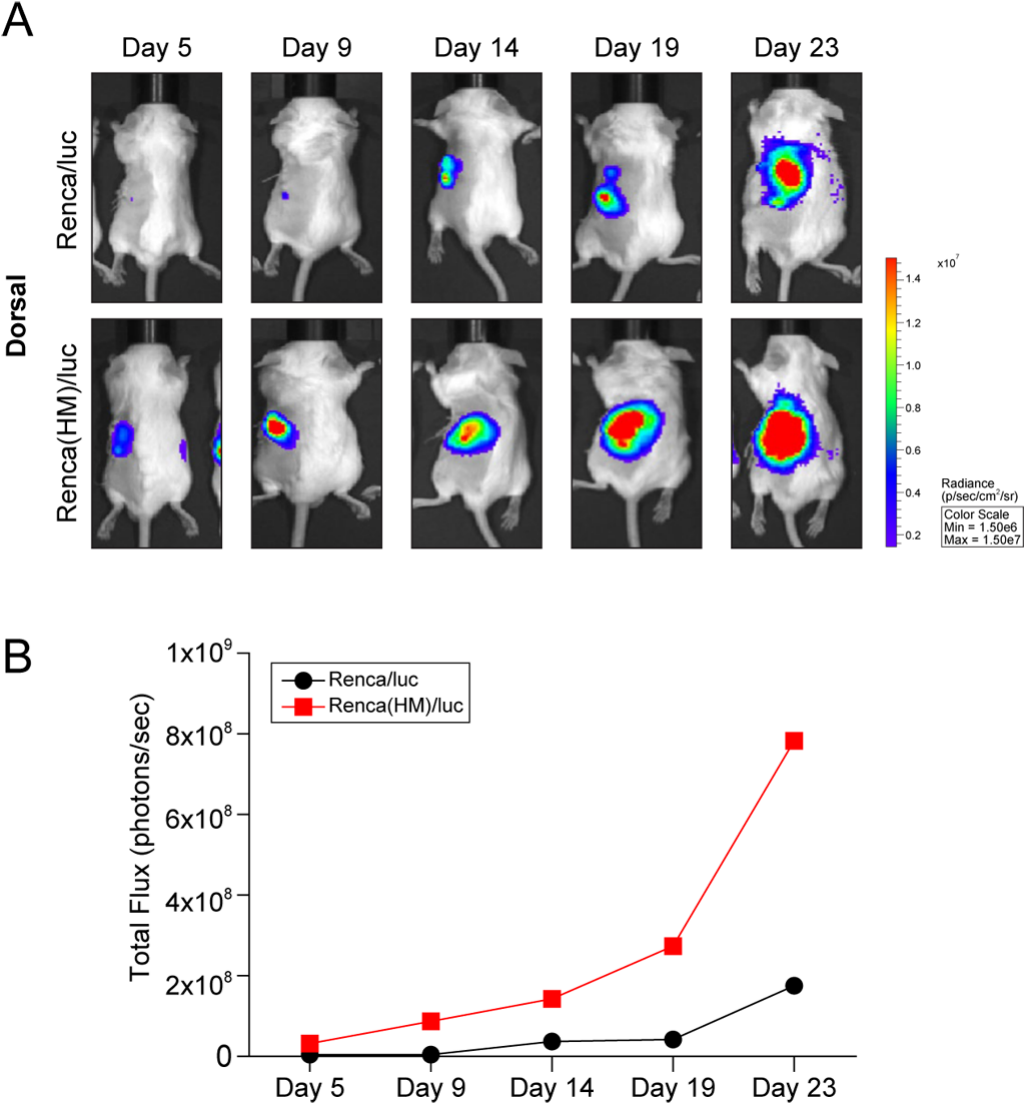
**Bioluminescent imaging (BLI) of orthotopically implanted Renca cells and spontaneous lung metastases.** (A) Representative bioluminescent images of BALB/c mice following intrarenal injection of 1×10^5^ Renca/luc or Renca(HM)/luc cells. (B) Higher BLI signal intensity (higher tumor burden) on day 5 following Renca(HM)/luc cell implantation (relative to day 9 following Renca/luc cell implantation) (*n*=2 for each day 5, 9, 14, 19, and 23 post implantation, total *n*=10 for Renca/luc and Renca(HM)/luc, respectively).

After Renca/luc cell implantation, all mice developed renal tumors and lung metastases. The *in vivo* imaging system (IVIS) longitudinally tracked tumor burden (Fig. 1A). BLI signal intensity was higher in Renca(HM)/luc cell-implanted mice than in Renca/luc cell-implanted mice (Fig. 1B). BLI signal intensity was higher in Renca(HM)/luc-cell-implanted mice (0.24×10^8^±0.07×10^8^ photons/sec, *n*=3) than in Renca/luc-cell-implanted mice (0.03×10^8^±0.01×10^8^ photons/sec, *n*=3) with significance (*P*=0.030) on day 5 post implantation. This difference was more pronounced over time with the BLI signal intensity of 23 post implantation of Renca(HM)/luc cell-implanted mice (9.81×10^8^±4.45×10^8^ photons/sec, *n*=12) higher than in Renca/luc cell-implanted mice (2.22×10^8^±0.49×10^8^ photons/sec, *n*=12) with significance (*P*=0.043). The post-implantation survival interval was calculated at a mean of 23 days.

**Fig. 2.**
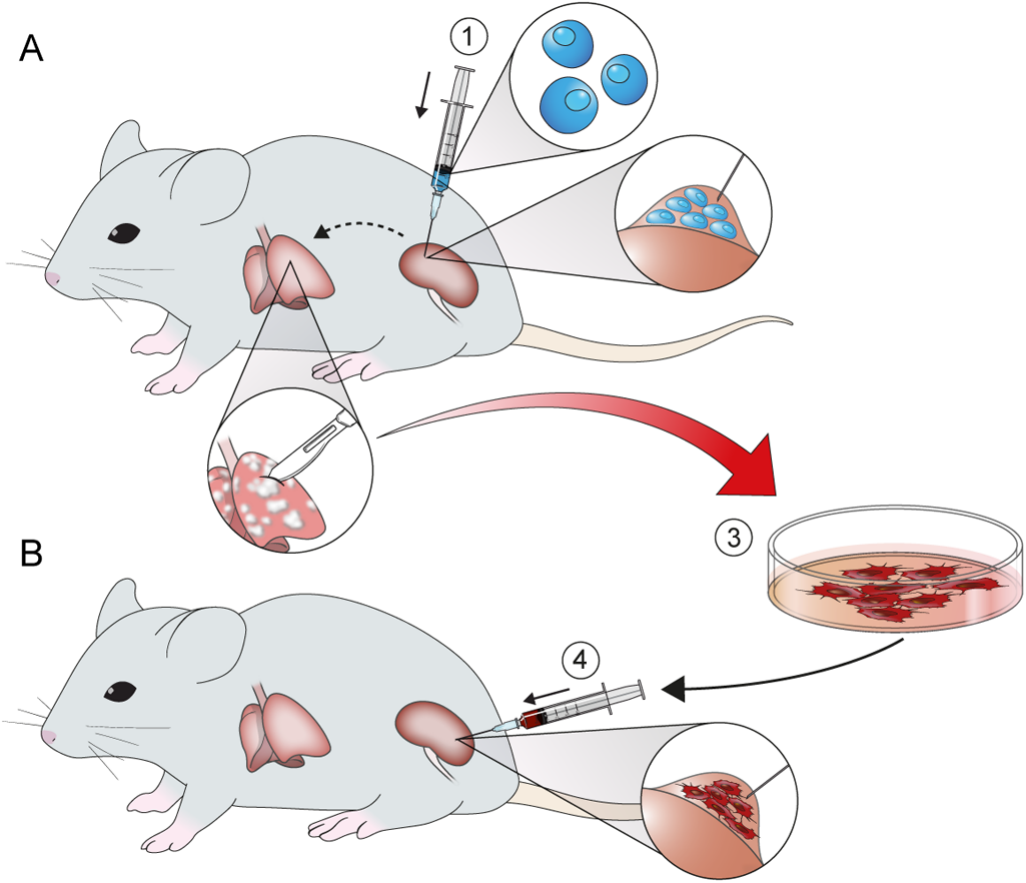
**Schematic representation of the *in vivo* selection process.** Cell lines were intrarenally injected (orthotopic implantation) in mouse (A), followed by isolation of tumor cells from pulmonary metastases for expansion culture and reinjection in another mouse (B). (1) Initial Renca/luc cell implantation. (2) Isolation of metastatic tumor cells from pulmonary lesions. (3) *In vivo* selection of the highly pulmonary metastatic cell subpopulation, termed Renca(HM)/luc. (4) Final reimplantation of Renca(HM)/luc cells.

**Fig. 3.**
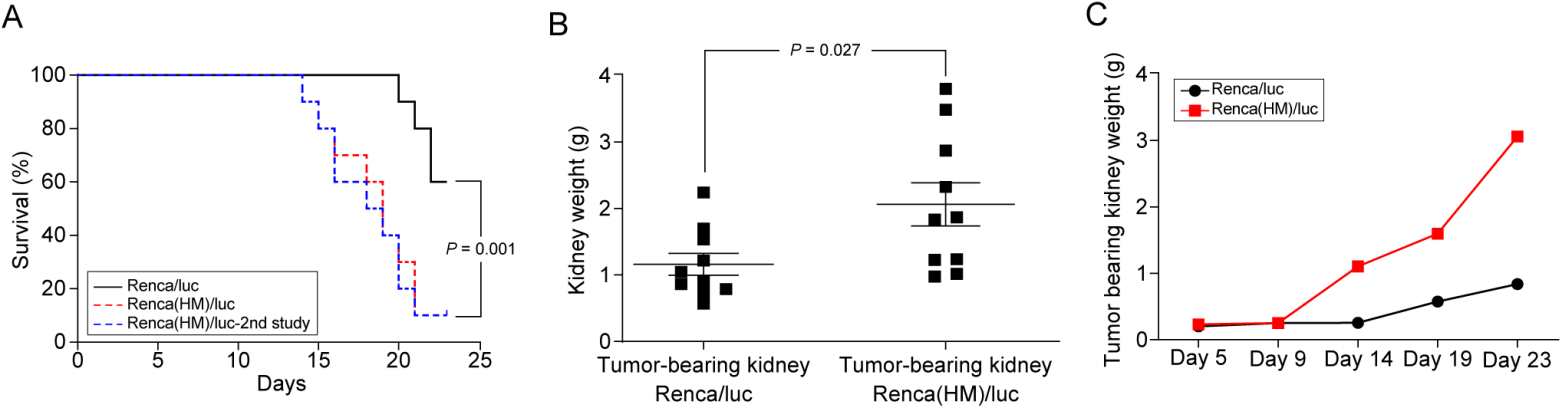
**Progression rate of primary renal tumors and lung metastatic lesions.** (A) Kaplan–Meier survival curves, indicating significantly shorter survival intervals for Renca(HM)/luc cell-implanted mice than for Renca/luc cell-implanted mice (*n*=10 for Renca/luc and Renca(HM)/luc). The dotted line represents the Kaplan–Meier survival curve following final Renca(HM)/luc cell-reimplantation(2nd study, *n*=10). (B) Excised tumor-bearing kidney weights of Renca/luc cell-implanted mice relative to those of Renca(HM)/luc cell-implanted mice (*n*=10 for Renca/luc tumor-bearing, and Renca(HM)/luc tumor bearing kidneys). (C) Higher weight of the tumor-bearing kidney (more rapid tumor growth) following Renca(HM)/luc cell implantation (relative to Renca/luc cell implantation) (*n*=2 for each day 5, 9, 14, 19, and 23 post-implantation, total *n*=10 for Renca/luc and Renca(HM)/luc, respectively).

Because tumor cell populations are heterogeneous, displaying differential metastatic capabilities (Fidler and Kripke, 1977), an *in vivo* selection approach was used to isolate a highly metastatic (HM) subpopulation of Renca/luc cells, termed Renca(HM)/luc, with unique characteristics underlying their preference for pulmonary metastasis (Fig. 2). After Renca(HM)/luc cell implantation, mice demonstrated a significant decrease in survival: Renca(HM)/luc cell-implanted mice were euthanized 18.6±2.9 days post-implantation, whereas Renca/luc cell-implanted mice were euthanized 22.3±1.1 days post-implantation (*P*=0.001, *n*=10 for each group; Fig. 3A). To assess the degree of metastatic enhancement induced by consecutive *in vivo* selection, Renca(HM)/luc cells were again isolated and reimplanted into a third set of mice, but no superior metastatic potential was observed after this second passage. Specifically, no significant difference in survival was observed (relative to the original Renca(HM)/luc cell-implanted mice; *P*=0.785, *n*=10 for each group; Fig. 3A). Furthermore, the number of observed pulmonary metastatic lesions did not differ significantly between the original Renca(HM)/luc cell-implanted mice (172.6±38.2) and Renca(HM)/luc cell-reimplanted mice (183.9±39.0; *P*=0.521, *n*=10 for each group).

Original Renca(HM)/luc cell implantation also increased renal tumor growth rate, resulting in larger tumors and a higher tumor burden. Renca(HM)/luc cell-implanted mice demonstrated a significantly higher tumor burden (2.0±1.0 g) than Renca/luc cell-implanted mice (1.2±0.5 g; *P*=0.027, *n*=10 for each group; Fig. 3B). This difference was observed by day 14 and became more pronounced over time (Fig. 3C). No difference in weight of the tumor-bearing kidney was observed by day 5 post implantation {0.22±0.02 g (Renca/luc, *n*=3), 0.22±0.01 g [Renca(HM)/luc, *n*=3]; *P*=1.000}, but significant differences were observed on day 23 post-implantation {1.17±0.33 g (Renca/luc, *n*=12), 2.33±0.50 g [Renca(HM)/luc, *n*=12]; *P*=0.029}.

**Fig. 4.**
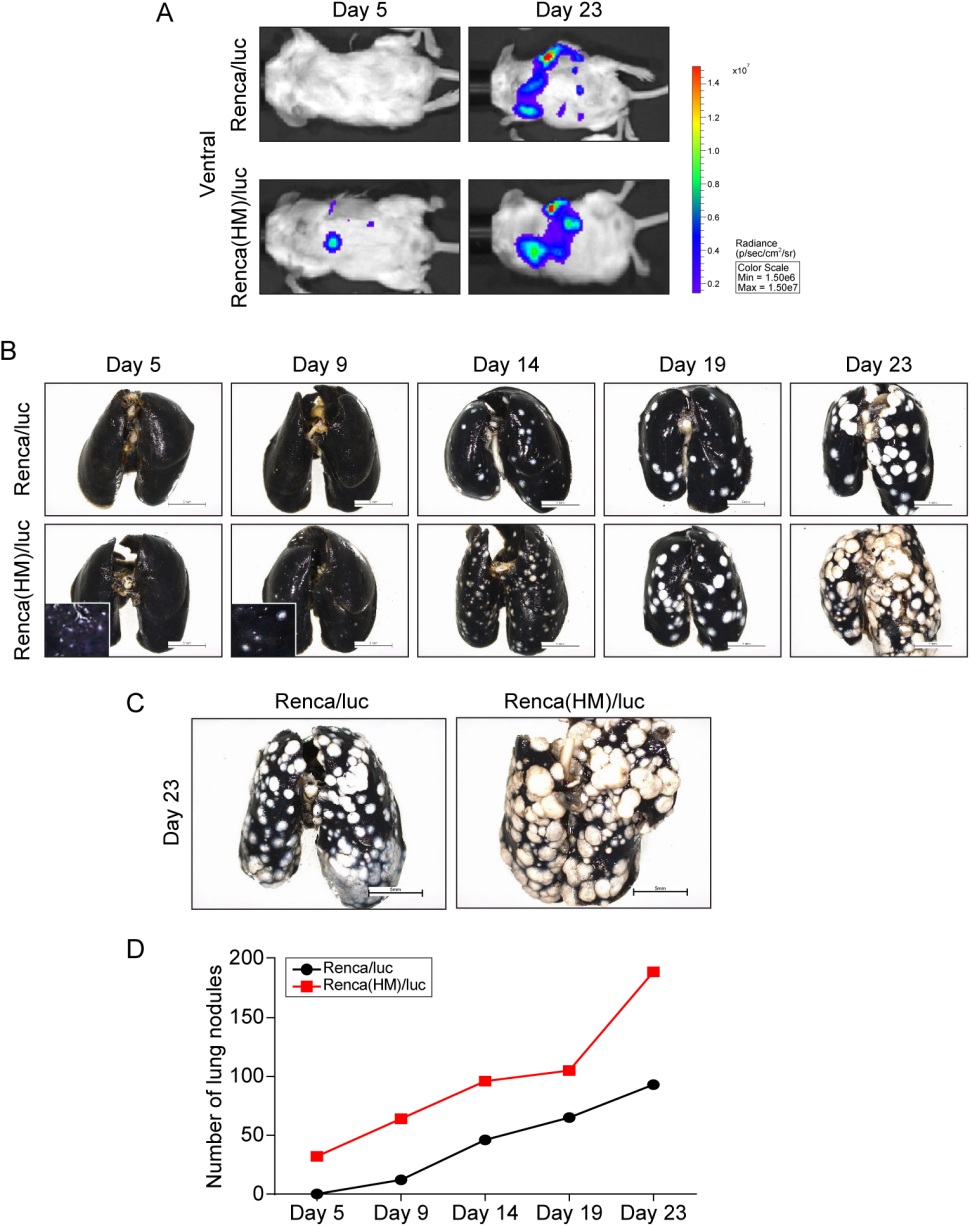
**Spontaneous lung metastasis following intrarenal implantation of highly pulmonary metastatic Renca(HM)/luc cells.** (A) Bioluminescent imaging demonstrating early (day 5) lung metastatic lesions in Renca(HM)/luc cell-implanted mice versus no signal in Renca/luc cell-implanted mice. (B) Number of pulmonary metastatic lesions over time, demonstrating more lesions in Renca(HM)/luc cell-implanted mice than in Renca/luc cell-implanted mice. (C) Pulmonary metastatic lesions visualized via India ink-insufflation in Renca/luc cell-implanted mice and Renca(HM)/luc cell-implanted mice on day 23 post-implantation. Scale bars: 5 mm. (D) Number of pulmonary metastases over time, demonstrating significantly more lesions in Renca(HM)/luc cell-implanted mice than in Renca/luc cell-implanted mice [*n*=2 for each day 5, 9, 14, 19, and 23 post-implantation, total *n*=10 for Renca/luc and Renca(HM)/luc, respectively].

Finally, Renca(HM)/luc cell implantation resulted in a shorter interval to development of pulmonary metastases and increased the number of lung metastatic lesions (relative to Renca/luc cell implantation). Pulmonary metastasis was first observed via IVIS in Renca(HM)/luc-cell-implanted mice 5 days post-implantation, at which time Renca/luc cell-implanted mice exhibited no pulmonary metastases (Fig. 4A). Pulmonary metastasis was confirmed by India ink insufflation (Fig. 4B,C). The number of lung metastases on day 23 post implantation in Renca(HM)/luc cell-implanted mice (172.6±38.2) was significantly higher than that in Renca/luc cell-implanted mice (90.1±22.8; *P*<0.001, *n*=10 for each group). Pulmonary metastases developed more rapidly in Renca(HM)/luc cell-implanted mice, with a maximum growth rate difference on day 19 post-implantation (Fig. 4D).

## DISCUSSION

Despite the existence of multiple mRCC treatment modalities, overall survival remains low (under 2 years for the majority of patients), and no consensus has been achieved regarding an optimal treatment regimen (Czarnecka et al., 2014; Dutcher et al., 2020). Therefore, the development of more effective mRCC therapeutic interventions is required, which necessitates translationally relevant pre-clinical mRCC animal models, especially for pulmonary metastatic disease, as this is the most common site of RCC metastasis (Motzer et al., 1996). Therefore, we employed *in vivo* passage of the Renca cell line to develop a relevant immunocompetent BALB/c murine RCC model that efficiently develops pulmonary metastases. This model recapitulates an important hallmark of advanced human RCC (development of distant pulmonary metastases originating from a primary renal tumor) and offers the significant advantage of using a syngeneic tumor cell line to evaluate tumor progression and regression in an immunologically intact animal (James et al., 2012, 2014; Norian et al., 2012).

Few mRCC animal models have been developed (Murphy et al., 2017; Strube et al., 2010), and no other group has reported the development of a highly efficient pulmonary metastatic model. Since efficiency is a desirable model characteristic to produce time-efficient and cost-effective animal experiments, we used *in vivo* passage to select a highly metastatic cell subpopulation to overcome the long implantation-metastasis interval of the parental Renca/luc cell line. Moreover, an ideal model exhibits minimal variation between mice with respect to tumor progression. *In vivo* selection also helps address the heterogeneity inherent in cancer cell populations, including differential growth rates and metastatic potentials (Fidler and Kripke, 1977). In the present study, consecutive *in vivo* selection [reimplantation of Renca(HM)/luc cells] demonstrated that a single round of selection is sufficient to produce Renca(HM)/luc cells with relatively maximal metastatic potential. Thus, *in vivo* selection has facilitated the development of a murine model exhibiting a short implantation-metastasis interval as well as equivalent rates of cancer development between mice, which is suitable for pre-clinical therapeutic testing. While two other studies have used the *in vivo* selection approach to develop highly bone metastatic RCC models (Kang et al., 2003; Strube et al., 2010), the present study is the first to apply this methodology to developing a highly pulmonary metastatic RCC model.

A luciferase-expressing tumor cell line makes non-invasive longitudinal tracing and measurement of *in vivo* tumors feasible, representing a further advantage over the common orthotopic implantation model (in which tumor growth cannot be visualized in real time) as well as the subcutaneous implantation tumor model (in which tumor growth may only be measured by calipers). In addition, the subcutaneous tumor model does not exhibit metastasis to distal organs; the highly pulmonary metastatic model presented herein facilitates the elucidation of the pathological mechanisms underlying the metastasis of primary RCC to the lungs. Moreover, the present study demonstrates clear visualization of early pulmonary metastasis via BLI. Non-invasive detection of pulmonary metastases would facilitate determination of ideal treatment initiation times during experiments.

Numerous efforts to improve the survival of patients with mRCC have been made, which lead to enhanced survival in this era of targeted therapies and immunotherapies (Dizman et al., 2020; Tippu et al., 2020). Currently there exist several target agents of mRCC including vascular endothelial growth factor (VEGF) tyrosine kinase inhibitors, anti-VEGF monoclonal antibodies, and mammalian target of rapamycin (mTOR) inhibitors (Dizman et al., 2020; Tippu et al., 2020). Moreover, modern immunotherapies have been recently developed which target against PD-1, PD-L1, or CTLA-4, which disable the ability of tumor cells to evade the immune system without any significant side effects, especially in mRCC (Motzer et al., 2018, 2019; Rini et al., 2019). Therefore, we have focused on increasing the efficacy of modern immunotherapies in mRCC patients. We are currently under evaluation of clinical benefit of oncolytic virus which would increase sensitivity to PD-1 and CTLA-4 targeted immunotherapies, comparing with conventional targeted therapies including VEGF tyrosine kinase inhibitor and mTOR inhibitor using the model demonstrated here.

Furthermore, numerous genetic biomarkers have been developed, however, none of these markers have been clinically used in practice. Therapeutic and prognostic biomarkers would guide treatment of mRCC patients in determining the most effective therapeutic options. Therefore, we are planning on future studies that focus on unraveling the clinically feasible biomarkers of mRCC using this model.

There are few limitations of this study. First, due to the limitation of the number of mice used, only the small number of mice was used to compare between Renca/luc and Renca(HM)/luc cell-implanted mice group during the follow-up, euthanizing two mice per each day 9, 14, 19, and 23 post-implantation and two mice on day 5 post-implantation. Increasing the number of mice per each day would increase the total number of mice tenfold. Therefore, we allocated the minimum number of two mice in each day except day 5, in order to calculate statistical significance, and planned re-experiment when there is discrepancy between two mice. However, there was no discrepancy between two mice in each day and group. Second, due to the small number of mice in each group, statistical significance could not be calculated in some variables.

In conclusion, given the current increase in appreciation for the utility of anticancer immunotherapy, the need for appropriate pathophysiologically and clinically relevant animal models has increased concomitantly. The syngeneic, orthotopic, highly pulmonary metastatic RCC murine model presented herein is clinically relevant and highly efficient, exhibiting renal tumors that spontaneously produce pulmonary micrometastases as early as 5 days post-implantation. It will facilitate both the elucidation of biological mechanisms underlying pulmonary metastasis and the evaluation of multiple mRCC treatment modalities, including targeted therapies and immunotherapies.

## MATERIALS AND METHODS

### Animals

Animal experiments were conducted in accordance with the Guide to the Care and Use of Laboratory Animals approved by the Association for Assessment and Accreditation of Laboratory Animal Care and the National Institutes of Health guidelines. The experimental protocol was approved by the Institutional Animal Care and Use Committee (IACUC) of Yonsei University Health System (IACUC No. 2019–0151), following guidelines specified by the Institute of Laboratory Animal Resources Commission on Life Sciences National Research Council in the USA.

A total of 52 adult male BALB/c mice (Orient Bio Inc., Seongnam, GyeongGi-Do, Korea) aged 6–7 weeks, were maintained in clean animal facilities at Yonsei University Health System. Mice were housed five to a cage, with *ad libitum* access to autoclaved food, water, and bedding. Mice were euthanized when they became moribund. Among 52 mice, total 30 mice with 10 mice in each group [*n*=10 for Renca/luc, Renca(HM)/luc, and Renca(HM)/luc-reimplanted, respectively] were measured for survival, tumor growth, BLI signal intensity and number of lung metastasis at the end-of the study which was set as the time point of 23 days (Fig. S2A). For the measurement by the time points, a total of 20 mice with ten mice per each group of Renca/luc and Renca(HM)/luc (Fig. S2B) were used and two mice euthanized for each day 5, 9, 14, 19, and 23 post-implantation to measure BLI signal intensity (Fig. 1B), tumor-bearing weights (Fig. 3C), and number of lung metastasis (Fig. 4D) to observe the tendency according to the time not to compare statistical significance.

### Cell lines

Initially described in 1973, the Renca cell line was derived from a spontaneously arising murine renal adenocarcinoma; it is syngeneic in BALB/c mice and grows unimpeded in such animals (Murphy and Hrushesky, 1973). Cells were grown in RPMI 1640 supplemented with 10% fetal bovine serum (FBS; Gibco, Waltham, MA, USA), and 1% penicillin-streptomycin (Gibco). Cells were incubated in 5% CO_2_ at 37°C.

To induce stable expression of red fluorescent protein (RFP) and firefly luciferase (luc), Renca cells were transfected using a commercially available lentiviral vector containing luc 3 and RFP genes under control of an inducible suCMV promoter (Amsbio, Cambridge, MA, USA). Luciferase and RFP were bicistronically expressed as individual proteins from a single mRNA, mediated by a 2A peptide to ensure equimolar expression of both proteins. Additionally, a puromycin resistance marker was expressed under an RSV promoter to facilitate dual selection of successfully transduced cells via both RFP signal detection and puromycin resistance.

Renca/luc cells were grown to sub-confluence, harvested using 0.25% trypsin, resuspended in complete RPMI 1640 medium, and centrifuged to remove the supernatant. A hemocytometer was used to count viable cells, and volume was adjusted using Hank's Balanced Salt Solution (HBSS) to yield a concentration of 2×10^6^ cells/ml. The suspension was aspirated into a glass syringe coupled to a 22-gauge needle (both Hamilton, Bonaduz, GR, Switzerland) for orthotopic renal injection (implantation).

### Orthotopic implantation

Orthotopic implantation was performed as previously described (Murphy et al., 2017). Briefly, mice were anesthetized using intraperitoneal instillation of 30 mg/kg zoletil and 10 mg/kg rompun. A surgical anesthetic plane was confirmed via the toe-pinch test. The left flank was shaved and sterilized using a povidone-iodine solution, a 1 cm vertical incision was made using surgical scissors, and the dermis was separated from the peritoneum. The spleen and kidneys were identified through the peritoneum, both visually and by palpating the mouse from below. A 50 µl aliquot of cell suspension (corresponding to 1×10^5^ cells) was slowly injected, the needle was held in place for 5–10 s to minimize cell backflow, and the incision was closed after the needle was withdrawn. Post-operatively, animals were allowed to recover in a clean cage equipped with a warming pad.

### Serial passage to establish a highly pulmonary metastatic cell line subpopulation

Three weeks post-implantation of Renca/luc cells, mice were sacrificed, lungs were harvested, and pulmonary metastases were precisely harvested with the assistance of fluorescence microscopy. Pulmonary metastatic lesions were dissociated to form a single-cell suspension using the gentleMACS™ Tumor Dissociation Kit (Miltenyi Biotec, Auburn, CA, USA), according to the manufacturer's instructions. Tumor cells were purified from this suspension using a Tumor Cell Isolation Kit (Miltenyi Biotec), according to the manufacturer's instructions. This pulmonary metastatic cell subpopulation was termed Renca(HM)/luc and was maintained in RPMI 1640 medium supplemented with 10% FBS prior to again being intrarenally implanted, including subsequent isolation of pulmonary metastases (following an identical procedure to that described above). After each implantation, the interval to metastasis, tumor progression, and the interval to moribundity or demise were monitored.

### Bioluminescent imaging

To detect Renca/luc tumor cell dissemination, non-invasive, whole-body BLI was applied—using a charge-coupled device camera (Roper Scientific, Inc./Photometrics, Tucson, AZ, USA)—on days 5, 9, 14, 19, and 23 post-implantation. After anesthesia, mice received intraperitoneal instillations of luciferin (0.1 ml of a 30 mg/ml solution in sterile phosphate-buffered saline (PBS)). After 10 min, mice were placed in the BLI system and tumor burdens were evaluated in terms of photons of light emitted per second within a defined region of interest.

### Organ harvesting and evaluation

Mice exhibiting pulmonary metastatic lesions were sacrificed for organ harvesting. Prior to excision, the degree of pulmonary metastasis was assessed by insufflating the lungs with India ink. After excision and prior to weighing, extraneous connective tissue was removed from the tumor-bearing kidney, contralateral kidney, and lungs. Lungs were then immediately fixed in Fekete's solution, followed by manual counting of lung surface metastatic lesions under a dissecting microscope.

### Statistical analysis

Survival, tumor-bearing kidney weights, BLI signal intensity and number of pulmonary metastatic lesion comparisons on day 23 post-implantation employed *n*=10 or 12 mice per group, while tumor-bearing kidney weights, and BLI signal intensity comparisons on day 5 post-implantation employed *n*=3 per group. All results are expressed as the mean±standard deviation (s.d.). Student's *t*-test was used to compare continuous variables, and Fisher's exact test was used to compare categorical variables. Kaplan–Meier survival analysis (using the Log-Rank test) was performed to compare survival probabilities. Statistical analyses were performed using SPSS version 23.0 (IBM Corp., Armonk, NY, USA) and GraphPad Prism version 8.0 (GraphPad Software, Inc., La Jolla, CA, USA). All statistical tests were two-tailed, and *P*-values <0.05 were considered significant.

## Supplementary Material

Supplementary information
