# Supplementary material for: Development of a highly pulmonary metastatic orthotopic renal cell carcinoma murine model
Source: Biol Open. 2021 Apr 20;10(4):bio058566. doi: 10.1242/bio.058566 (PMC8084570; doi:10.1242/bio.058566)
Supplement: Supplementary information [file biolopen-10-058566-s1.pdf]

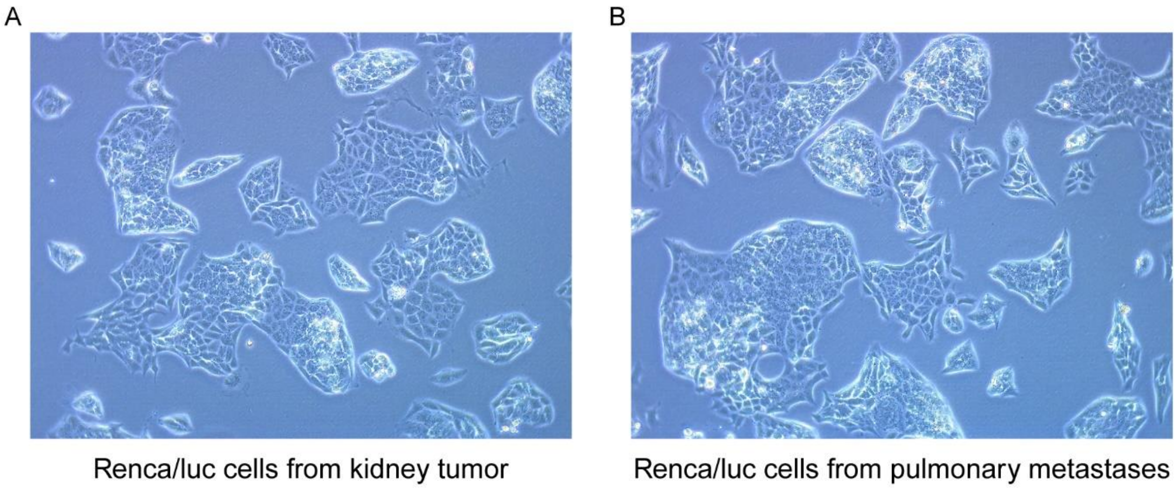


**Figure S1. Cell morphology of Renca/luc cells A.** Cells from kidney tumors. **B.** Cells from pulmonary metastases

Biology Open • Supplementary information

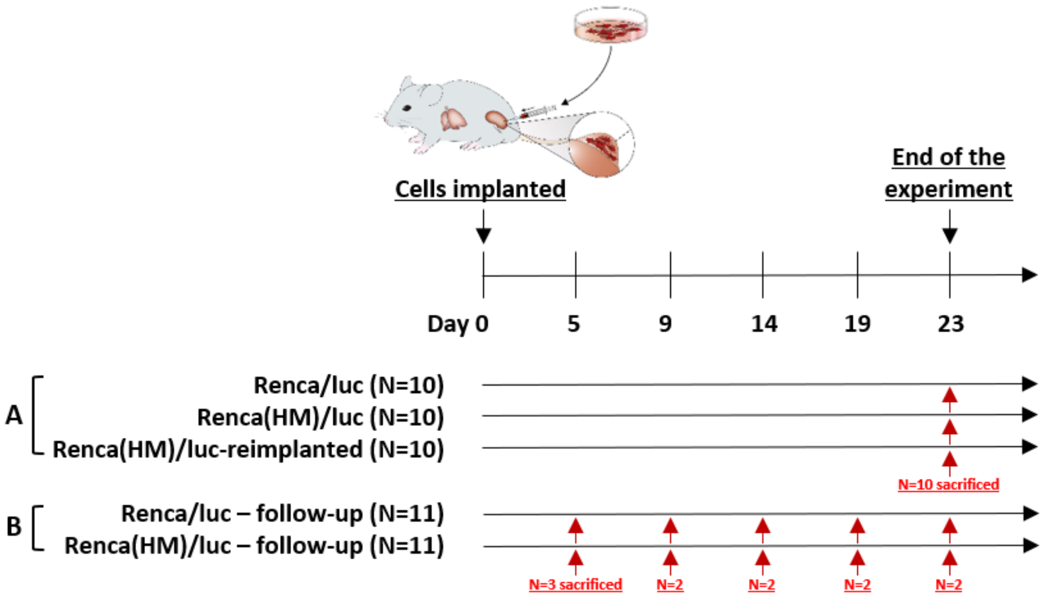


**Figure S2. Schematic representation of mice groups used in this study A.** 3 groups of mice sacrificed until the end of the study (n=10 for Renca/luc, Renca(HM)/luc, and Renca(HM)/luc-reimplanted, respectively). **B.** 2 groups of mice sacrificed with each time points to measure the tendency (2 mice sacrificed for each day 9, 14, 19, and 23 post-implantation and 3 mice sacrificed for day 5, total n=11 for Renca/luc and Renca(HM)/luc, respectively)

Biology Open • Supplementary information
